# Older Patients' Verbal Communication in Interactions With Primary Care Staff: A Qualitative Systematic Review and Meta‐Ethnography

**DOI:** 10.1111/hex.70355

**Published:** 2025-08-28

**Authors:** Rebecca Goulding, Peter Bower, Tom Blakeman, Sarah Peters

**Affiliations:** ^1^ Division of Musculoskeletal and Dermatological Sciences, School of Biological Sciences, Faculty of Biology, Medicine and Health The University of Manchester Manchester UK; ^2^ NIHR School for Primary Care Research, School of Health Sciences, Manchester Academic Health Science Centre The University of Manchester Manchester UK; ^3^ Manchester Centre of Health Psychology, School of Health Sciences, Manchester Academic Health Science Centre The University of Manchester Manchester UK

**Keywords:** aged, health communication, meta‐ethnography, patient involvement, primary healthcare, professional–patient relations, qualitative research

## Abstract

**Background:**

Communication between patients and staff is a crucial component of safe and effective healthcare. As people age, they have more consultations and these become more complex. As such, older people may be more likely to experience gaps and breakdowns in communication.

**Objective:**

To develop a better understanding of older people's communication in interactions with primary care staff and the barriers to and enablers of this.

**Search Strategy:**

Four databases were searched. Search terms covered the sample (older people), domain (verbal communication in healthcare interactions), context (primary care) and research type (qualitative research).

**Data Extraction and Synthesis:**

A meta‐ethnographic approach was followed by one researcher, with input from the wider team. Twelve studies were included. Details of the designs, participants, methods and results were extracted. Data were synthesised through reciprocal translation, and a line of argument was developed.

**Main Results:**

Barriers to communication were found in relation to raising and addressing concerns. Barriers arose from patient perceptions of their role, the nature of their relationship with staff, patient and staff perceptions of responsibility and reluctance to broach a topic, individual factors such as memory, how staff respond when concerns are raised and the degree of patient involvement in consultations. Potential enablers of communication were preparation and support from family or peers.

**Conclusions:**

Synthesising the existing qualitative literature on older people's communication in healthcare interactions enabled the identification of barriers and enablers that can be used to inform the development of an intervention to improve communication with primary care staff.

**Patient or Public Contribution:**

By identifying healthcare communication as the most important topic for the patient safety of older people with multiple long‐term conditions in primary care and setting the scope of the review, patients and the public were involved in the design of the study. Four public contributors (older people with multiple long‐term conditions and carers of older people with multiple long‐term conditions) attended regular meetings, provided their reflections on the study findings and supported the interpretation of the data.

## 1 Introduction

Communication between patients and staff is a crucial component of safe and effective healthcare [1, 2]. It is important for diagnosis and treatment, and is associated with physical and psychological outcomes and satisfaction with care [3, 4, 5]. Communication problems can leave patients feeling disempowered, devalued and unsafe [6, 7]. Communication is at the centre of most medical malpractice claims [8].

In primary care, consultation rates increase with age, and most consultations with those aged 65+ are considered complex [9, 10]. In the context of ageing and other diagnosed conditions, communication problems are more likely to arise, and health concerns may be normalised rather than recognised and responded to [11, 12]. As such, older people may have the most to gain from improved healthcare communication.

Research on, and efforts to improve, healthcare communication often focus on staff rather than patients' behaviour. Measures of how patient‐centred a consultation is generally only assess the contribution of staff [13], and approaches developed to improve communication, such as Connecting, Assessing, Responding and Empowering (CARE) [14] and Background, Affect, Trouble, Handling and Empathy (BATHE) [15], focus on helping staff connect with and elicit information from patients. However, when exploring why some patients express opinions and concerns, and ask questions more than others, Street et al. found these behaviours were predominantly patient‐initiated [16].

The importance of patient involvement in healthcare interactions is increasingly recognised [17] and is thought to be ‘*the biggest and yet least understood’* (p. 7) domain for patient safety [18]. While some interventions to improve patients' healthcare communication increase expressing concerns, asking questions and overall participation, others have had no impact [19, 20]. The reasons for this are not fully understood. The authors of one systematic review [19] noted very few interventions were grounded in theory, and further research was required to strengthen the evidence base to better understand patient perspectives on and barriers to communication.

Very few systematic reviews have focused on older people's healthcare communication. A 2007 review [21] found just three interventions aimed at improving older people's verbal communication with primary care staff. One successfully supported patients to provide information and ask questions [22]. Another, designed to empower patients and increase their participation, found trained patients were more satisfied with the interpersonal aspects of interactions [23]. However, there was potential for bias in these studies due to issues with sample size and allocation concealment [21]. In an effort to identify more recent interventions, a subsequent review [24] focused on older people with multiple long‐term conditions. A further three studies, which aimed to increase patient involvement in decision‐making during primary care consultations, were found. Despite providing training to patients through workshops and one‐to‐one coaching, and encouraging patients to think about what they wanted to discuss, none of these studies reported patient communication behaviours as an outcome. Thus, despite their potential, there remains a lack of interventions for older people, developed from a strong theoretical and evidence base, and high‐quality research in this area [19, 21, 24].

The study presented here is part of a wider programme of research designed to develop an intervention to empower older people, improve communication in primary care and reduce risks to patient safety. A theory‐, evidence‐ and person‐based approach is being followed, which emphasises the use of qualitative research to ensure interventions are responsive to needs [25]. A first step is to establish the existing evidence base and develop a better understanding of the relevant behaviour. Information on older people's healthcare communication can be derived from a range of sources, including observations of interactions and the recollections of those involved; including the patients, staff and other individuals who may be present.

### 1.1 Objectives

To review the existing qualitative literature to develop a better understanding of older people's verbal communication in interactions with primary care staff. Specifically, to describe the nature of this communication and the barriers to and enablers of communication in this context.

## 2 Methods

Various approaches to synthesising qualitative data exist. Meta‐ethnography facilitates conceptual innovation, with authors bringing studies together to arrive at an understanding that is more than the sum of its parts [26, 27, 28]. It is suitable for use with a disparate literature, as was expected to be found through this review [27].

Meta‐ethnography has seven steps [26]. These steps, though followed sequentially, overlap and have been conducted in different ways [26, 29]. For this review, the steps were operationalised as outlined in Table 1. The eMERGe guidance for reporting a meta‐ethnography and the Preferred Reporting Items for Systematic reviews and Meta‐Analyses (PRISMA) 2020 statement for reporting the search have been followed [30, 31].

**Table 1:** The steps of a meta‐ethnography.

Step		How operationalised
1. Get started		Determine that a synthesis would be useful and define objectives
2. Decide what is relevant		Search for and identify potentially relevant reports, refine inclusion and exclusion criteria, develop and run a comprehensive search, screen records and reports and select studies
3. Read		Read and re‐read the reports, extract data and appraise reports
4. Determine how the studies are related		Identify similarities and differences between the studies and how the extracted data might be brought together
5. Translate the studies into one another		Systematically compare the studies and organise the extracted data, grouping together data that are conceptually similar
6. Synthesise the translations		Identify overarching concepts or conceptual categories and bring these together in a line of argument, determining what these add to our understanding
7. Express the synthesis		Present the products of the synthesis (narrative, tables and figures)

### 2.1 Search Strategy

A comprehensive search strategy was developed for four databases. These were searched, from inception to 31 December 2020, via the Ovid (MEDLINE, Embase and PsycINFO) and EBSCO (CINAHL) platforms. The searches were restricted to the English language as resources for translation were not available. Backwards searching, from reference lists of included papers, was also conducted.

The SPIDER (Sample, Phenomenon of Interest, Design, Evaluation, Research type) tool was used to develop the search strategy [32]. While the PICO (Participants, Interventions, Comparisons, Outcomes) tool and its variants are generally recommended by leading review organisations, the I, C and O are not always relevant or appropriate to research questions being answered through a qualitative review [32, 33, 34]. One study comparing the use of SPIDER and PICO identified a potential problem with the use of separate blocks of search terms for design and research type, as relevant papers did not always contain both [35]. Therefore, these have been grouped together. Search terms for evaluation have not been included. Instead, the phenomenon of interest has been broken down into domain and context (see below).

Search terms for the sample and domain were adapted from a relevant Cochrane review [21], and those for the context and research type were adapted from published database filters. The final search is presented in Supporting Information 1.

In line with the meta‐ethnographic approach, the inclusion and exclusion criteria were refined and updated during step two when deciding what was relevant to the review objectives [26, 36]. The original inclusion and exclusion criteria can be found in the registered protocol for this study [37]. The final criteria are detailed below.

### 2.2 Inclusion and Exclusion Criteria

#### 2.2.1 Sample

To centre this review on older people and to manage its scope, studies were only included if they had older patient participants. Although 65 is commonly used as the lower boundary for an older population, the relationship between age and health varies [38]. For example, multiple long‐term conditions can occur 15 years earlier among those living in deprived areas [39]. Thus, an older sample was defined as one where all participants were aged 50+ and the majority were aged 65+. Studies were included if informal carers (family members or friends) or primary care staff (clinical and/or administrative) were recruited alongside older patient participants.

As the focus was on communication between patients and staff, populations likely to require carer support during healthcare interactions were excluded. This included studies where any of the patient participants were care home residents, had significant cognitive impairment or severe mental health problems or were unable to provide informed consent. Studies were also excluded if any of the participants were proxies for or exclusively provided care to people who met the exclusion criteria.

#### 2.2.2 Phenomenon of Interest—Domain

Studies were included if the aims and objectives focused on patients' verbal communication in healthcare interactions. This included evaluations of interventions to improve or studies of the barriers to and enablers of such communication.

Studies were excluded if they focused on a broader topic, where verbal communication in interactions was only one aspect (e.g., studies of access to care or trust in care providers). Studies were also excluded if they focused on communication difficulties because of an impairment, or due to differences in language, ethnicity or culture.

#### 2.2.3 Phenomenon of Interest—Context

Studies were included if the interactions took place in primary care. That is, settings in which ‘*first‐contact, continuous, comprehensive, and coordinated care [is] provided to populations undifferentiated by gender, disease, or organ system*’ (p. 1129) [40].

The focus was specifically on settings where general/family medicine is provided. Thus, studies were excluded if they concerned ophthalmology, dental or community mental health services. Studies were also excluded if they focused on transitions of care or concerned home or social care (as opposed to community‐based primary care).

#### 2.2.4 Research Type

Studies that used qualitative methods of data collection and analysis were included. Mixed‐methods studies were included if the qualitative aspect was separate and substantial.

Studies were excluded if the only qualitative data were from questionnaires, surveys or medical records, or if the method of analysis was content analysis.

### 2.3 Screening and Data Extraction

Duplicate records were identified and removed, and then the titles and abstracts of the remaining records were screened. Where potentially relevant records were dissertations or conference abstracts, the authors were emailed to see if the research had been published. Full texts of all potentially relevant journal articles were screened. Where needed to determine eligibility for inclusion, authors were contacted for more information.

Included texts were read and re‐read, and data extracted. Where studies used mixed methods or were part of a wider study, data extraction focused on the qualitative aspects. A data extraction template, used in a previous study, was adapted [1]. The template included sections on general information (the study design, location and objectives), participants (number, characteristics and recruitment), methods (of data collection and analysis) and results, with the results being the interpretations of, that is, quotes from the authors (second‐order constructs) as opposed to quotes from the study participants (first‐order constructs) [26, 41]. Results were extracted according to the authors' identified themes or subheadings, where present. In other cases, the aims of the study or phrases were used as themes.

The data extraction template also included a quality assessment section, adapted from the Critical Appraisal Skills Programme (CASP) Qualitative Checklist [42]. Reports were categorised as key, satisfactory, unsure, fatally flawed or irrelevant to the research question [43]. The full data extraction template is presented in Supporting Information 2.

Screening, data extraction and appraisal were conducted by one researcher (R.G.), who sought input from the wider team when deciding what was relevant. Uncertainties, including articles categorised as ‘unsure’, were discussed with a second researcher (S.P.) and resolved. No articles were categorised as fatally flawed or irrelevant.

### 2.4 Data Translation and Synthesis

Although there were differences in study topics, designs and methods, each study identified communication behaviours, barriers and/or facilitators. The data were viewed as interrelated and therefore possible to add together through a reciprocal rather than refutational translation [26]. As such, the studies were arranged chronologically and worked through in order, systematically comparing more recent studies to each of those published before [44]. Initially, a table was created to summarise and compare the characteristics of included studies. The extracted results data were read, re‐read and organised into tables to show the concepts identified within and across the studies [44]. Here, a concept is defined as ‘*a meaningful idea that develops by comparing particular instances’* (p. 7) [41].

This initial translation of the studies was read and re‐read to identify commonalities and connections within and between the concepts and bring these together into conceptual categories. These conceptual categories are interpretations of the authors' interpretations (third‐order constructs) [41]. Finally, these conceptual categories were brought together into a single model or line of argument to go beyond the individual studies and develop a better understanding of older patients' communication in primary care [26, 45].

Throughout the translation process, to preserve the meaning of the data, the wording used by authors of the included studies was retained [45]. Although conducted by one researcher (R.G.), each step was discussed with the wider research team, including patient and public contributors, within regular meetings. Discussion of the initial translation led to further refinement and the development of the line of argument.

## 3 Results

### 3.1 Overview and Appraisal of Studies

**Figure 1:**
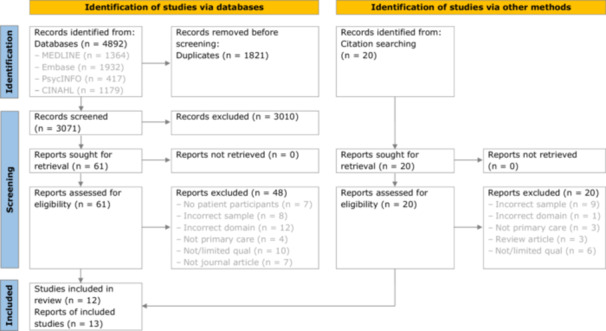
PRISMA 2020 flow diagram.

After duplicates were removed, 3071 records were identified through database searches, of which 61 were selected for full‐text review. Thirteen reports, discussing 12 studies, were deemed eligible for inclusion (see Figure 1). All included reports were published after 2000, and more than half were published after 2015, suggesting this is a recent body of research in which interest is growing.

A summary of the included studies is presented in Table 2. All studies were conducted in higher‐income, predominantly English‐speaking countries. In eight of the studies, data were collected through interviews or focus groups. The four remaining studies were observational, with data collected through audio‐ or video‐recording healthcare interactions [46, 47, 48, 49]. All studies reported barriers to and/or facilitators of communication. The observational studies also detailed the nature of communication (its content and context) between patients and staff.

**Table 2:** Summary of included studies.

Report	Population	Population characteristics	Setting and location	Study design	Aim/objective	Method of data collection	Method of data analysis
Towle et al. (2003) [54]	9 older people who attended an intervention workshop	Age (inclusion criteria)†: 65+ Education: > 60% post high school	‘*Talking with your doctor’* intervention workshop delivered in North Vancouver, Canada	Qualitative study	To explore ‘*the possibilities of enhancing patient participation through a community‐based intervention’* (p. 231)	Brief semi‐structured interviews, approx. 2 months after the workshop	Grounded theory analysis of transcripts
Wittink et al. (2006) [53]	48 older people who self‐identified as being depressed (27 rated as depressed by their physician, 21 not)	Age (mean): 73 physician rated as depressed, 77 not Gender: 36 female, 12 male Education: 18 less than high school	Non‐academic primary care practices in Baltimore, Maryland, the United States	Integrated mixed‐methods study (primarily qualitative)	‘*to understand aspects of the physician‐patient relationship … that may influence the way patients communicate about depression’* (p. 303)	Semi‐structured interviews	Constant comparative method, derived themes from transcripts
Wallhagen and Pettengill (2008) [52]	Patients: 91 older people with an untreated hearing impairment	Patients: Age (mean, range): 73 (60–93) Gender: 52 male Education: 61 post high school	Primary care. Authors based in San Francisco, California, the United States	Part of a longitudinal qualitative and quantitative study	Qualitative aspect explored the effects on patients of not being asked about or screened for hearing loss	Interviews—patients and communication partners were interviewed separately	Constant comparative method
	Family/friends: The patients' ‘*communication partners’* (p. 37)						
Friedland and Rudman (2009) [55]	Patients: 79 older people (29 presenior drivers, 24 senior drivers and 26 senior ex‐drivers)	Age (mean, range): preseniors 60 (55–64), senior drivers 76 (66–92), ex‐drivers 82 (65–94) Gender: Majority female Education: Majority high school	Family physician practices in a large city in Ontario, Canada	Secondary analysis of data from a qualitative study	To explore: ‘*how [patients], physicians, and family members communicated, or failed to communicate, about driving’* (p. 13)	Focus groups, using separate guides for patients and staff	Secondary/additional in‐depth, inductive analysis of transcripts, formed themes
	Staff: 20 family physicians from a practice where at least 25% of patients are aged 65+	Gender: 10 female, 10 male					
Vannoy et al. (2011) [49]	Patients: 6 older people who discussed suicide in a video‐recorded medical visit	Age (mean, range)‡: 74 (67–88) Gender: 3 female, 3 male	3 primary care practices in the Southwest and Midwest, the United States	Qualitative study	To ‘*Identify patterns in physician‐patient communication regarding suicide’* (p. 1005)	Video‐recorded medical visits (385 visits were recorded—mental health was discussed in 84, suicide in 6 of the 84)	Thematic analysis of videos and transcripts
	Staff: 3 primary care physicians	Gender: 1 female, 2 male					
Lum et al. (2016) [46]	32 older people who participated in a group medical visit (GMV) intervention	Age (mean): 79 Gender: 19 female Education§: 1 less than high school, 3 high school, 24 post high school	A seniors clinic providing primary care in Aurora, Colorado, the United States	Pilot/feasibility study with qualitative aspect	Qualitative aspect focused on ‘*What aspects of advance care planning will older adults discuss in the GMV?’* (p. 128)	Audio‐recorded group medical visits	Inductive and deductive analysis of transcripts, constant comparison, identified themes
Lenzen et al. (2018) [47]	Patients: 5 older people, with whom practice nurses ‘*planned to discuss goals and agree on action plans’* (p. 390)	Age (mean, range): 81 (75–85) Gender: 3 female, 2 male Education: 2 less than high school, 1 high school, 2 post high school	Primary care in the Netherlands	Qualitative study	‘*to examine how practice nurses … set goals and formulate action plans with their elderly patients’* (p. 390)	Observed and video or audio‐recorded home visits (depending on patient approval)	Conversation analysis of ‘*episodes of goal setting and/or action planning’* (p. 391)
	Staff: 3 practice nurses working with older people						
Malta et al. (2018) [50]	Staff: 15 GPs 6 practice nurses	Gender: 7 female GPs, 8 male GPs, 6 female practice nurses	General practices in Australia	Qualitative study—part of a larger programme of research	To explore the barriers and enablers to discussions about sexual health between older people and primary care staff	Semi‐structured interviews	Inductive and deductive thematic analysis
Malta et al. (2020) [58]	Patients: 21 older people	Age (range): 60–90+ Gender: 9 female, 12 male					Thematic analysis, following a mixed etic/emic approach
Schöpf et al. (2018) [57]	Patients: 6 older people taking 5 or more medications	Age (mean): 75 Gender: 3 female, 3 male Education: 4 less than high school, 1 high school, 1 other qualification	A large primary healthcare centre in rural Germany	Qualitative study	To explore ‘*perceptions of communication about polypharmacy, medication safety and approaches for empowerment’* (p. 355)	Semi‐structured interviews	Framework approach to analyse transcripts
	Staff: 3 GPs/medical professionals	Gender: 3 male					
Waterworth et al. (2018) [51]	21 older people with 2 or more long‐term conditions, in receipt of nurse‐telephone communication	Age (average, range): 77 (66–90) Gender: 11 female, 10 male	6 general practices, from rural and urban areas of New Zealand	Qualitative study	To explore ‘*older people's experiences of nurse–patient telephone communication’* (p. 374)	Semi‐structured interviews	Thematic analysis ‘*informed by a constructivist grounded theory approach’* (p. 373)
Muscat et al. (2019) [48]	Patients: 20 older people	Age (mean): 75 Gender: 10 female, 10 male Education§: 5 high school or less, 5 post high school, 6 degree	5 general practices in Australia	Mixed‐methods analysis of secondary data	‘*to explore how decisions are made in … consultations … and examine how general practitioners … communicate evidence and integrate patient preferences’* (p. 880)	Video‐recorded consultations	Framework approach to thematically analyse transcripts
	Staff: 9 GPs	Gender: 2 female, 7 male					
Glaudemans et al. (2020) [56]	Patients: 22 older people with experience of advance care planning (ACP)	Age: 6 70–79, 12 80–89, 4 over 90 Gender: 11 female, 11 male Education‡: 8 less than high school, 8 high school, 5 post high school	Primary care in the Netherlands	Qualitative study	‘*To explore older people's and their families' experiences with ACP in primary care’* (p. 519)	Semi‐structured interviews	Inductive analysis of transcripts, grounded theory approach
	Family: 8 family members of the older people	Age (range): 40–79 Gender: 7 female, 1 male					

^†^ Lead author confirmed ‘*seniors*’ refers to people aged 65+.

^‡^ Missing data for 1 participant.

^§^ Missing data for 4 participants.

The types of demographic information reported for patient participants varied across the studies. Education was reported inconsistently or not at all [49, 50, 51]. However, it appeared most were high school educated, and more than half attended education post high school. Ethnicity was only reported for four studies [46, 49, 52, 53]. In those, the majority were White (54%–100%). Gender was not reported for one study, and in another it was only noted that most were female [54, 55]. Across the remaining studies, there were similar numbers of female (144, 53%) and male (128, 47%) patient participants.

Two studies included family members or friends [52, 56]. Six included staff [47, 48, 49, 50, 55, 57]. Specifically, General Practitioners (GPs)/physicians (4 studies), practice nurses (1 study) or both (1 study). There were similar numbers of female and male staff participants. However, when broken down by staff group, there were more male (28, 60%) than female (19, 40%) GPs/physicians, and more female (7, 78%) than male (2, 22%) practice nurses.

Following quality appraisal, all studies were rated as satisfactory. Study designs were considered appropriate, although they were not always justified within the reports. All reports lacked some methodological information, particularly in relation to recruitment and data collection or the interpretation and presentation of results (see Table 3). More thorough reporting was seen in more recent studies [48, 51, 56, 57].

**Table 3:** Appraisal of included studies.

Study	Are the following aspects of the study specified, justified and appropriate?				Are enough data presented to support interpretations and conclusions?
	Aims and objectives	Design	Recruitment and data collection	Data analysis	
Towle et al. (2003) [54]	Yes	To some extent	To some extent	To some extent	To some extent
Wittink et al. (2006) [53]	Yes	Yes	To some extent	To some extent	To some extent
Wallhagen and Pettengill (2008) [52]	Yes	To some extent	To some extent	To some extent	To some extent
Friedland and Rudman (2009) [55]	To some extent	Yes	To some extent	To some extent	To some extent
Vannoy et al. (2011) [49]	Yes	To some extent	To some extent	Yes	To some extent
Lum et al. (2016) [46]	To some extent	To some extent	To some extent	Yes	To some extent
Lenzen et al. (2018) [47]	Yes	To some extent	To some extent	To some extent	To some extent
Malta et al. (2018 and 2020) [50, 58]	Yes	To some extent	To some extent	To some extent	To some extent
Schöpf et al. (2018) [57]	Yes	To some extent	To some extent	Yes	Yes
Waterworth et al. (2018) [51]	Yes	Yes	Yes	Yes	To some extent
Muscat et al. (2019) [48]	Yes	Yes	To some extent	Yes	Yes
Glaudemans et al. (2020) [56]	Yes	Yes	To some extent	To some extent	Yes

*Note:* Appraisal based on the CASP Qualitative Checklist [42].

### 3.2 Results of the Synthesis

Through the synthesis, two key aspects of communication during healthcare interactions were identified: raising concerns and addressing concerns. Barriers that can hinder these aspects of communication are described, and potential enablers are reported. Finally, a line of argument synthesis is presented, in which these results are brought together to tell the ‘story’ of communication between older people and primary care staff.

#### 3.2.1 Raising Concerns

Barriers to health concerns being raised include patients' perceptions of their role (being a ‘good’ patient), relationships with staff (too close or not close enough), perceptions of responsibility for raising concerns (each waiting for the other to initiate), reluctance to raise health concerns (staff caution, patient embarrassment or fear, lack of time) and other/individual factors (see Table 4).

**Table 4:** Translational table: Raising concerns.

Third‐order constructs	Second‐order constructs/data	
	Key theme(s)	Example data from each contributing report
Role	I'm a good patient [53]	‘Maintenance of a good rapport … was perceived to be important to get the best possible health care’ (p. 232) [54].
		‘The notion of the good patient is further manifested as a particular role that may be co‐constructed by the physician and patient … a role that is perhaps even expected’ (p. 305), ‘might inhibit any discussion of emotions without happy or positive content’ (p. 308), ‘the good patient … does not complain or burden their physician. Discussing emotional difficulty with the physician may be seen as unnecessary complaining’ (p. 308) [53].
		‘Patients did not tell their GPs about non‐adherence as they … did not want to be seen as incompliant’ (p. 357), ‘had to take the medication in order to be an obedient patient’ (p. 358) [57]
Relationship	Openness to ACP and trust [56]	‘Relationships that were too close could, in fact, be detrimental to patients revealing intimate details about their sexual lives’ (p. 810) [50].
		‘Friendliness of the GP and continuous care by the same GP promote an open discussion. Hindering factors are … lack of trust’ (p. 357) [57].
		‘Viewed the doctor as the expert and authority on all health matters and did not feel that seeing a nurse would be of any benefit’ (p. 378), ‘Some felt they could use this opportunity to raise any other concerns they may have. Having a prior relationship with the nurse and trusting them, influenced this decision’ (p. 379) [51].
		‘Lacking trust or negative previous experiences … with a GP or nurse could be a reason to be less open’ (p. 521) [56].
Responsibility	Dialogue concerning medication: whose responsibility? [57]	‘GPs stated it was a joint responsibility between themselves and their patients’ (p. 809), ‘almost all GPs left it primarily to the patient’ (p. 809) [50].
		‘Older patients generally felt their doctors should be the ones to begin such discussions’ (p. 43), ‘if sexual health was “normalised” by being routinely addressed in regular or annual check‐ups, this would diffuse some of the discomfort associated with such conversations’ (p. 45) [58].
		‘The plan is not always or sometimes only partly reviewed’ (p. 358), ‘GPs are seen by patients and GPs as having primary responsibility’ (p. 360) [57].
		‘Provided an opportunity for the patient and their GP to review each medication's purpose and continued use. However, this did not happen’ (p. 883) [48].
Reluctance	Issues contributing to reluctance to discuss driving [55]	‘Time (e.g. doctor always busy)… Reluctance to bother the doctor’ (p. 232) [54].
		‘Physicians focus mostly on the physical issues and tend to ignore emotional ones’ (p. 306), ‘patients feel any discussion of emotional issues will lead to a referral to a psychiatrist’ (p. 306), ‘patients are clearly not bringing up emotional issues because they may believe their physician will not be interested’ (p. 308) [53].
		‘Many participants were not fully aware of their loss or denied its significance, often for years, especially when they had concerns about having to wear a hearing aid. The importance of having a primary care provider who specifically addresses hearing loss and initiates the testing process was often noted’ (p. 40) [52].
	Getting and providing feedback in the ‘right way’ [55]	‘Physicians … talked about the potential negative impact of raising the topic of driving on relationships’ (p. 17), ‘seemed to avoid thinking about—and discussing—what life would be like when they were no longer able to drive’ (p. 18), ‘importance of continuing to feel that they were in control of the ultimate decision’ (p. 18), ‘Physicians also stressed the importance of providing feedback in a nonconfrontational way’ (p. 19), ‘expectation that feedback will be given if they fail to recognize their own limitations’ (p. 20) [55].
		‘Would only ask about sexual health directly if it was relevant to the patient's presenting complaint’ (p. 809), ‘most preferred a patient‐directed consultation’ (p. 809), ‘allowed practice nurses the time to build rapport and gently explore sensitive lines of questioning’ (p. 809), ‘practice nurses were generally perceived as approachable’ (p. 810) [50].
		‘Reticent to bring up … for fear of judgement’ (p. 45), ‘noted feeling shamed … as though admitting to sexual interest was wrong and/or inappropriate in later life’ (p. 45) [58].
		‘Might even conceal information’ (p. 355), ‘all patients declared that they spoke openly with their GPs’ (p. 357), ‘Hindering factors are … embarrassment’ (p. 357), ‘it is necessary to have courage’ (p. 357), ‘did not mention over‐the‐counter (OTCs) or herbal drugs as they felt uncomfortable revealing their use’ (p. 357), ‘it is often necessary to actively ask patients about their real medication intake’ (p. 358) [57].
		‘Older people may not raise certain concerns e.g. feeling low or depressed with health professionals’ (p. 374), ‘The feeling of being heard by the nurses and being afforded the time to be heard, was particularly valued’ (p. 378) [51].
		‘Open to ACP if they felt a need to arrange important matters in order to prevent unwanted future situations’ (p. 521), ‘Negative thoughts regarding GPs' time or interest … appeared to make respondents less open’ (p. 521) [56].
Other	Barriers to sexual health discussions with older patients [50]	‘Memory (e.g. forgets intent to talk about something)’ (p. 232) [54].
		‘Barriers to discussion included age and gender disparity’ (p. 810) [50].
		‘Patients did not tell their GPs about non‐adherence as they … thought the GP was already aware of it’ (p. 357) [57].

Patients want to be seen as good by, and maintain rapport with, healthcare staff [53, 54]. As such, they attempt to enact a role they perceive as being expected of them [53]. Good patients do not complain and are compliant [53, 57]. Thus, enacting this role can inhibit discussion of, for example, negative emotions and non‐adherence [53, 57].

Established relationships and the perception of staff as friendly, competent and trustworthy can motivate patients to view interactions as an opportunity to raise concerns and enable open communication [51, 56, 57]. However, being too close could hinder communication and prevent patients from revealing information about themselves [50, 57].

Perceptions of whose responsibility it was to raise a health concern varied. GPs saw themselves and patients as having joint responsibility regarding sexual health, but, in practice, few initiated such discussions [50]. Patients thought GPs should take responsibility and that it would reduce their discomfort if sexual health discussions were part of routine interactions [58]. Even when both parties agreed responsibility lay with staff, as was the case for medication reviews, comprehensive discussions were infrequent and communication opportunities missed [48, 57].

In some instances, staff were reluctant to broach a topic deemed irrelevant to the reason for the interaction [50]. Staff wanted to protect the patient–provider relationship by allowing interactions to be patient‐directed and discussing topics in a non‐confrontational way [50, 55]. Patients expected and noted the importance of staff initiating discussions about topics they themselves may not recognise the need for, such as hearing loss, the ability to continue driving and advance care planning (ACP) [52, 55, 56]. Patients expressed a preference for these discussions to take place in the context of preventing ‘*unwanted future situations’* (p. 521) and when they can be in control of decision‐making [55, 56].

Patients' reluctance to raise health concerns stemmed from embarrassment, shame, fear of judgement, concerns about treatment or wanting to avoid thinking about the future [52, 55, 57, 58]. Thus, they may need courage to raise a concern [57]. Patients also felt staff tended to ignore or were not interested in mental health and believed raising this topic would result in them being ‘*turfed’* (p. 308) to a specialist [53]. Patients, who otherwise felt able to speak openly with staff, may feel uncomfortable sharing information about their use of non‐prescribed or herbal medicines. However, staff awareness that patients may conceal such information led to them asking patients about their ‘*real medication intake’* (p. 358) [57].

Time was also a factor, as GPs were perceived as being busy and patients were reluctant to bother them or raise a topic they would not have time to discuss [54, 56]. However, practice nurses had more time with each patient and considered themselves to be approachable and able to gently broach sensitive topics [50, 51].

Other factors that acted as barriers to concerns being raised related to individual characteristics, such as the age of GPs and the gender of patients [50]. Patients may also forget to mention something or not recognise the need to do so [54, 57].

#### 3.2.2 Addressing Concerns

Raising concerns is only part of the story. Concerns also need to be addressed. Barriers to addressing concerns include the relationship between patients and staff (friendliness over focus), response from staff (dismissed and discouraged) and degree of patient involvement (persuading passive patients or encouraging active participation) (see Table 5).

**Table 5:** Translational table: Addressing concerns.

Third‐order constructs	Second‐order constructs/data	
	Key theme(s)	Example data from each contributing report
Relationship	Superficial Pattern: Engaging in Chitchat [49]	‘A strong pull for the patient to reassure the physician that he is okay’ (p. 1009), ‘suicide is acknowledged, and care is expressed but there is a quick move to chitchat…. This results in a superficial and misleading connection’ (p. 1009), ‘abrupt transitions away from the topic’ (p. 1009) [49].
		‘Discussions about subsidiary topics such as “small talk” … took up a considerable proportion of the consultation’ (p. 881‐2) [48].
Response	Argumentative Pattern: Life's Not That Bad [49]	‘Providers discounted the importance of hearing difficulties’ (p. 40) [52].
		‘Strives to convince the patient that suicide is unwarranted’ (p. 1005), ‘lack of overt empathic validation’ (p. 1007), ‘despite efforts to be heard, this patient finds the door is being held shut’ (p. 1009) [49].
		‘Felt unable to discuss sexual health with older patients when there were more complex or urgent issues’ (p. 809) [50]
		‘Participants affirmed that sex was important to them’ (p. 43), ‘Clinician attitudes often discouraged them from pursuing the conversations further’ (p. 43), ‘expressing ageist attitudes, by signalling to older patients that sex was not an acceptable topic for them to be concerned about at their age’ (p. 44) [58].
		‘Health issues that seemed important to the patient were minimalised and deferred or disregarded’ (p. 882), ‘concerns … were commonly dismissed’ (p. 883), ‘at several points … expressed confusion … and worry’ (p. 884), ‘the GP did not respond to this concern’ (p. 884) [48].
Involvement—passive	Considering the patient: Preferences and practicalities [48]	‘Rather than explicitly rejecting that option … he states that giving his wife a hug is the best thing he can do’ (p. 1009), ‘does tell the patient she wishes to increase the dose of his antidepressant medication’ (p. 1009) [49].
	The passive patient [47]	‘Declined the solutions offered’ (p. 391), ‘the problem discussions were closed with no action plan agreed upon’ (p. 391) ‘enquires whether the patient has ever considered a specific action’ (p. 392), ‘the question is followed by an alternative … which reverses the preference structure’ (p. 392), ‘remains silent … and thus displays resistance’ (p. 393), ‘interrupts … with a well‐prefaced turn … signalling non‐straightforwardness’ (p. 393), ‘pursue acceptance of available services’ (p. 393), ‘did not ask questions about what a patient wanted to change or achieve’ (p. 394), ‘treated patients as passive agents (persons who are not capable of actively engaging in solving the problem in question)’ (p. 394), ‘there was a lack of a shared view regarding the patient's problem’ (p. 395), ‘did not frequently explore the patient's perspective’ (p. 395) [47].
		‘They differed regarding the amount and the type of information they wanted’ (p. 359), ‘they would neither question the GP's decision nor ask for more information as they trusted that the doctors would know what they were doing’ (p. 359) [57].
		‘Expressed confidence in competence of their GPs based on their past experiences and what appeared to be long‐standing relationships’ (p. 882), ‘dismissed the request for more information and prescribed … without further explanation’ (p. 882), ‘the provision of patient choice as a method of involvement was limited, with GPs steering patients towards the treatment option that they thought was in their patients' best interest, by offering it as the only option’ (p. 883), ‘GPs often seemed to infer that their patients had low preferences for information … and to make assumptions about the degree of involvement the patient needed’ (p. 884), ‘the patient corrected the GP's assumption that she did not need or want to know’ (p. 884), ‘few GPs explicitly elicited patient preferences’ (p. 884), ‘immediate response of the patient was to accept the change’ (p. 884), ‘did not comment at all on the recommendations of their GPs’ (p. 884) [48].
Involvement—active	From passive patient to active agent [47]	‘Reflecting back the patient's statements’ (p. 1009), ‘Immediately validates the patient by acknowledging his thoughts’ (p. 1009) [49].
		‘Approached him as a person who is capable of actively engaging in solving his problem’ (p. 391), ‘Even more exceptional … is that after a short pause … the [practice nurse] … positions the patient as an active participant’ (p. 394) [47].
		‘One exception was a rare example…’ (p. 883) [48].
		‘If … a nurse or GP had explicitly explained reasons for ACP … respondents did not feel it had been too confronting’ (p. 521), ‘They were positive about the attention they received during these conversations, felt heard and more at ease’ (p. 522) [56].

The relationship between staff and patients could divert attention from the topic being discussed. Staff may acknowledge the concerns raised and express care but then transition away by engaging patients in ‘*chitchat’* (p. 1009) [48, 49]. Such interactions could be superficial, with small talk taking up considerable time [48, 49]. Vannoy et al. (2011) suggested that, in such relationships, patients may feel a need to reassure staff and say they are okay when they are not [49].

Observational studies showed patients raised concerns, sometimes repeatedly, during healthcare interactions [48, 49]. However, concerns could be ignored, not validated, discounted, dismissed or argued against [48, 49, 52]. This discouraged further patient involvement, and the topic was closed with no resolution [58]. Some concerns that were important to patients were deferred or disregarded by staff in the context of multiple health conditions [48, 58]. In these circumstances, staff prioritised the concerns they saw as more complex or urgent [50].

Alternatively, staff may respond to a concern by attempting to problem solve and suggesting a specific action or declaring what the treatment will be without actively engaging patients or seeking their perspective or preferences [47, 48, 49]. Staff appeared to assume patients would not want information about their options or to be involved in the decision‐making process [48]. Patient preferences differed, and although they rarely asked questions, when they did, these were not always answered [48, 57].

Suggestions from staff can be met with resistance from patients. Patients may communicate their uncertainty or disagreement by staying silent, indicating confusion or ‘*non‐straightforwardness’* (p. 393), mentioning an alternative action or declining [47, 49]. In response, staff may rephrase or elaborate on their suggestion to try and persuade patients to accept it. Such discussions end with no agreed plan [47]. In contrast, some patients would immediately accept without question a suggestion from a trusted care provider, particularly in the context of an established relationship [48, 57].

In what was described as exceptional and rare instances, patients' concerns were validated, and they were actively involved in action or care planning [47, 48]. This involved staff acknowledging and reflecting back what had been said, and then explaining the need for a plan and seeking patient input [47, 48]. Such discussions could result in an agreed plan and leave patients feeling heard and positive about the interaction [56].

#### 3.2.3 Potential Enablers

Studies, including the two reported interventions, provided insights into what may help improve communication. Suggestions focused primarily on empowering patients to raise concerns through preparation (making and using notes) and support (practical and social, from family and peers) (see Table 6).

**Table 6:** Translational table: Potential enablers.

Third‐order constructs	Second‐order constructs/data	
	Key theme(s)	Example data from each contributing report
Preparation	Facilitating sexual health discussions with older patients [58]	‘Need to prepare; present information effectively; communicate expectations; express concerns, ask questions’ (p. 232), ‘Futility (e.g. inability to make a difference in the relationship)’ (p. 232), ‘most barriers to communications … were attributed to the doctor (outside of patient's control)’ (p. 232), ‘the difficulty of changing an established pattern of communication’ (p. 232) [54].
		‘Some individuals had not thought much about what was important to them, while others had’ (p. 129) [46].
	Supporting patients' engagement: how? [57]	‘GPs and practice nurses were willing to embrace resources that could provide appropriate education and information for themselves and their older patients’ (p. 810) [50].
		‘An online resource in the form of a website could be beneficial’ (p. 45), ‘Most felt [a checklist] could be both useful and acceptable’ (p. 45), ‘could help circumvent the difficulties they and their doctors experienced’ (p. 46) [58].
		‘To bring medication plans, relevant documentation from other providers and diaries’ (p. 359), ‘Patients thought that notes of questions and information would prevent them forgetting … and considered that they were more self‐confident when they came to the consultation on an informed basis’ (p. 360) [57].
Support	Supporting patients' engagement: how? [57]	‘Members of smaller cohorts … tended to mention fewer personal examples, ask fewer question, and discuss a smaller range of advance care planning experiences and topics than those in larger groups’ (p. 130) [46].
		‘Relatives as an important means of support’ (p. 360), ‘motivate her to go to the doctor and address difficult topics’ (p. 360) [57].
	Roles of family in ACP [56]	‘Recollection … appeared to be easier … if family had been present’ (p. 521), ‘felt … it would burden their family too much or felt they could talk more freely without family present’ (p. 522) [56].

Interventions could and should encourage patients to prepare for interactions, gather information, and have notes or relevant documentation to hand to help them feel confident, communicate effectively and reduce reliance on memory [54, 57]. Resources, such as a website or checklist, to enable patients to prepare and avoid difficulties, were welcomed by both patients and staff [50, 58]. However, it was acknowledged that it can be difficult to change established communication behaviours, and some patients perceived efforts to change communication as futile and outside their control [54].

Family members were considered important sources of support and could motivate patients to raise topics they found difficult to broach [57]. The presence of family could also improve patients' recall of interactions. However, some were concerned about being a burden or, for example, when discussing ACP, felt freer to talk when alone [56]. Lum et al. (2016) found that the presence of peers could facilitate discussions about ACP, with those in larger groups asking more questions and discussing a wider range of topics than those in smaller groups [46].

#### 3.2.4 Line of Argument

**Figure 2:**
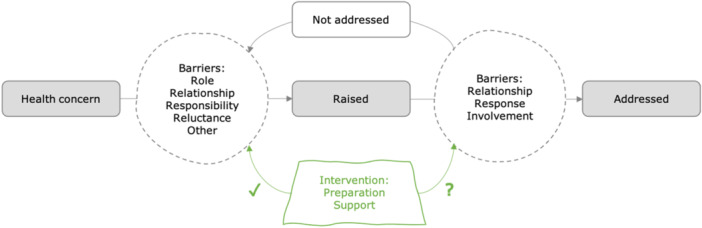
The story of communication between older people and primary care staff.

Bringing together the results from the included studies enabled the identification of two key aspects of communication that can be hindered during healthcare interactions between older people and primary care staff: raising concerns and addressing them. If a concern is raised but not addressed, the first set of barriers will need to be overcome once again (see Figure 2).

There is an overlap between the barriers to raising and addressing concerns, for example, with the patient–staff relationship being relevant to both. There are also differences that may require different interventions to help overcome. The potential enablers of communication put forward and tested in the included studies focused on empowering patients to raise concerns through preparation and support. From this literature, it is less clear if these or other interventions could empower patients to overcome the barriers to addressing concerns. Descriptions of barriers to addressing concerns often referred to the behaviour of staff, such as them engaging patients in small talk, dismissing or deferring concerns and failing to explore concerns and possible actions with patients. However, the role of patients could also be of influence. When patients accept a passive role, no agreement is reached, but when encouraged to take an active role, patients and staff can work together and find a mutually acceptable course of action.

## 4 Discussion

### 4.1 Summary of Results and Comparison to Other Literature

We set out to investigate older people's verbal communication in interactions with primary care staff by conducting a meta‐ethnography and bringing together relevant qualitative literature [26, 27]. Two key aspects of communication that can be hindered during these interactions were identified: raising concerns and addressing concerns. Barriers were identified in relation to each, as well as perceptions of what communication behaviours are acceptable and how communication might be enabled.

Both patients and staff were concerned about raising a topic that could threaten their relationship, and may wait for the other to raise it. Although no topic was identified as being off limits, mutual reluctance led to some concerns not being raised. Delays in raising concerns can threaten patients' health and safety and have the potential to cause rather than avoid animosity in a relationship [55, 59].

When raised, it is important to ensure concerns are addressed. When patients feel they have not been listened to, and their concerns are minimised or dismissed, this can threaten their sense of safety and impact their future communication behaviours, including whether or not to consult with a care provider again [7, 59].

The two potential enablers of communication that were identified (preparation and support) focused on empowering patients to raise concerns. It is less clear what strategies, if any, could help them overcome barriers to addressing concerns, which this review has found to predominantly relate to the behaviour of staff. However, reviews of existing interventions have shown that it is possible to empower patients to take a more active role and ask more questions during consultations and that these patients obtain more information [19, 21]. One intervention, which also had staff‐ and organisation‐focused components, found patients were more likely to report receiving care related to their priorities [60]. However, it was not clear which aspect of the intervention led to this change.

Behaviour is complex and influenced by a range of factors, including assumptions, beliefs, needs, intentions, social cues and interpersonal skills [61]. Communication behaviour is likely to be habitual and difficult to change, especially among patients who see the course of an interaction as being outside of their control [54, 62]. Developing a better understanding of the barriers to and potential enablers of communication is an important first step for being able to develop an effective behaviour change intervention [25, 61].

### 4.2 Strengths and Limitations

In bringing together studies covering a range of topics and using different methods of data collection and analysis, it was possible to simultaneously investigate the nature of communication between older people and primary care staff, and how this might be constrained or facilitated. While the steps in a meta‐ethnography are clearly outlined, how these steps are conducted, particularly how data are extracted, organised and brought together, varies [29, 44]. Lee et al. (2015) postulate this may be ‘*inevitable*’ given its interpretive and thus ‘*creative*’ nature [29]. If approached in a different way, the synthesis may have developed and been presented differently. However, the main findings and conclusions may not have changed [44].

The interpretive nature of meta‐ethnography and the need for repeated reading and translation of the included studies can mean this is not a suitable approach for dealing with a large literature [29, 44]. This was considered when refining the inclusion and exclusion criteria for the review. Thus, these were narrowed to focus on the reports most likely to be conceptually rich and make an appreciable contribution to the synthesis [36, 41]. After becoming familiar with the available literature, in a change to the original protocol, studies with no patient participants and those that used content analysis were excluded, and studies were only included if they were specific to primary care. As a result, the number of studies identified through this review was manageable. It is possible that further insights could have been gained from including a broader literature.

Although topics varied, in some respects the identified studies were relatively homogenous. They were conducted in developed countries, with patient participants who were predominantly White and educated to a high school level and beyond. Although the synthesis may provide a good understanding of communication for this population, it may not hold true for other populations. While this may reflect a lack of diversity in research, it is possible that a wider range of barriers and enablers would have been identified had the search extended beyond English language publications, or included studies that focused on differences due to language, ethnicity or culture. For example, within some cultures, people may be more likely to have a deferential approach to communicating with GPs that can result in certain health concerns not being raised [63].

The identified studies focused on communication between patients and GPs or nurses. No studies were found that included administrative staff, such as receptionists or practice managers. This could reflect a lack of research with such staff groups, a shortcoming of the search strategy, or the exclusion of studies that focused on access to care. The latter was outside the scope of this review but would be an interesting and important literature to investigate to identify barriers to and enablers of communication with primary care staff outside of and before consultations. Research, not specific to older people with MLTC‐M, has shown that patients sometimes view reception staff as gatekeepers despite their attempts to do what they can to help [64].

One experienced health services researcher, with a background in health psychology, led on all aspects of this study. Discussions with the wider research team, which included a GP and an expert in healthcare communication, influenced each step of the meta‐ethnography. Having multiple members of the team conducting steps such as screening, data extraction and translation may have further increased rigour and the credibility of the synthesis [29, 36]. Additionally or alternatively, the validity of the third‐order constructs and line of argument could have been tested by inviting authors of the included studies to review and provide feedback on these [45]. As Noblit and Hare (1988) note: ‘*Like all interpretations, a meta‐ethnography is but a “reading” of what is studied. Other readings are possible and are to be encouraged. However, all interpretations must be grounded in the texts to be synthesized*’ (p. 40) [26]. To ensure the analysis remained grounded in the included studies, the authors' words were retained throughout, and reports were revisited to ensure these had not been taken out of context [45].

### 4.3 Implications

The results from this review reveal that healthcare communication could be improved by supporting patients to prepare for interactions, raise their concerns and take an active role in discussions and decision‐making. However, established communication behaviours, rooted in patients' beliefs about their role in an interaction and the impact their behaviour could have on their relationship with staff, may be difficult to change. As such, patients may also need to be educated about and persuaded of the benefits of speaking up and being more involved in healthcare interactions. Thus, a patient‐focused intervention to improve healthcare communication would likely need to be multifaceted, addressing a range of barriers to communication and doing so in several ways. This is in line with Anderson and Sharpe's (1991) recommendation that such interventions should address a broad range of skills and behaviours [65].

Although this review was predominantly focused on the role of patients in their interactions with primary care staff, it also revealed aspects of staff behaviour that act as barriers to concerns being raised and addressed. As such, the results could also inform the development of or enhance existing staff‐focused interventions to improve communication and add further support to guidelines and policies that recommend staff involve patients in discussions about their care [17, 66]. For example, educating staff about the barriers to and enablers of healthcare communication and how their behaviour can influence the outcomes of interactions and patients' future help‐seeking behaviour could further motivate staff to use existing approaches (such as CARE) to better understand and respond to their patients' concerns [14]. System changes may also be required to ensure staff have time to employ such strategies.

### 4.4 Further Research

This review established the existing qualitative evidence base around and resulted in a better understanding of older people's verbal communication in interactions with primary care staff, represented through a line of argument or conceptual model that can be tested, critiqued and/or expanded through future research. As only 12 relevant studies were identified, the existing evidence base is small and has limitations. Additional primary qualitative research could be conducted to further investigate older people's healthcare communication and identify barriers and enablers that may be present for a more diverse range of older patients and in interactions between patients and different staff groups.

The studies included in this review collected data through interviews and focus groups, and observations of interactions between patients and staff. Most studies employed a form of thematic analysis. Only one of the observational studies used conversation analysis, which can be used to study ‘*how speakers take turns at talk; how talk is shaped by prior actions and shapes what follows it’* (p. 86) [67]. Thus, additional studies employing this method could reveal further insights about how the communication behaviours of staff influence those of patients and vice versa.

This review focused on studies that used qualitative methods of both data collection and analysis. However, some observational studies use quantitative coding schemes, such as the Verona Coding Definitions of Emotional Sequences (VR‐CoDES), to analyse data [68]. The VR‐CoDES distinguishes between cues and concerns from patients, that is, a suggestion (cue) or unambiguous expression (concern) of an unpleasant emotion, and explores how these are responded to by staff. Thus, a review encompassing or primary research using such methods may provide further insight into the nature of communication and usefully inform the development of interventions. As noted by Heritage and Maynard (2006), both conversation analysis and observational coding have limitations, but the learning obtained from using both approaches ‘*should result in a greatly enhanced view of the medical encounter’* (p. 360) [69].

## 5 Conclusion

By bringing together the existing qualitative literature, this review has expanded our understanding of barriers to and enablers of healthcare communication for older people. In doing so, it has provided important insights for the development of interventions to empower older people, improve their communication with staff and reduce risks to patient safety in primary care.

## Author Contributions

**Rebecca Goulding:** conceptualisation (lead), funding acquisition (lead), methodology (lead), investigation (lead), formal analysis (lead), visualisation (lead), writing – original draft preparation (lead), writing – review and editing (equal). **Peter Bower:** conceptualisation (supporting), funding acquisition (supporting), formal analysis (supporting), writing – review and editing (equal). **Tom Blakeman:** funding acquisition (supporting), formal analysis (supporting), writing – review and editing (equal). **Sarah Peters:** conceptualisation (supporting), funding acquisition (supporting), methodology (supporting), investigation (supporting), formal analysis (supporting), writing – review and editing (equal).

## Disclosure

The views expressed in this publication are those of the author(s) and not necessarily those of the NIHR, NHS or the UK Department of Health and Social Care.

## Conflicts of Interest

The authors declare no conflicts of interest.

## Supporting information


**Supplementary Material 1:** Search strategy.

**Supplementary Material 2:** Data Extraction Template.

## Data Availability

The data that support the findings of this study are available from the corresponding author upon reasonable request.
