# Supplementary material for: Older Patients' Verbal Communication in Interactions With Primary Care Staff: A Qualitative Systematic Review and Meta‐Ethnography
Source: Health Expect. 2025 Aug 28;28(5):e70355. doi: 10.1111/hex.70355 (PMC12392129; doi:10.1111/hex.70355)
Supplement: Supplementary file 1 — Supplementary Material 1: Search strategy. [file HEX-28-e70355-s001.docx]

## **SEARCH STRATEGY**

**Search strategy for the Ovid platform**

| Sample: Older people | |
| --- | --- |
| 1 | exp aging/ |
| 2 | ((old or older or aging or senior) adj3 (person or persons or people or adult$ or subject$ or patient$ or consumer$)).tw. |
| 3 | ((old or older or aging or senior) adj3 (service user$)).tw. |
| 4 | ((old or older or aging or senior) adj3 (men or male or males or women or female$)).tw. |
| 5 | (late life or elder$ or old$ age or geriatric or seniors).tw. |
| 6 | or/1-5 |
|  | |
| Phenomenon of Interest - domain: Patient verbal communication in interactions | |
| 7 | Communication/ |
| 8 | Doctor Patient Relations/ |
| 9 | Patient Education/ |
| 10 | Patient Participation/ |
| 11 | Consumer Participation/ |
| 12 | Patient-Centered Care/ |
| 13 | Decision Making/ |
| 14 | Advance Directives/ |
| 15 | Audiovisual Aids/ |
| 16 | Decision Support Techniques/ |
| 17 | ((patient$ or client$ or consumer$ or recipient$ or subject$ or service user$ or care?giver$ or care giver$ or carer$ or famil$) adj3 (communicat$)).tw. |
| 18 | ((patient$ or client$ or consumer$ or recipient$ or subject$ or service user$ or care?giver$ or care giver$ or carer$ or famil$) adj3 (verbal or talk or discourse$ or discussion$ or conversation$)).tw. |
| 19 | ((patient$ or client$ or consumer$ or recipient$ or subject$ or service user$ or care?giver$ or care giver$ or carer$ or famil$) adj3 (interact$ or relation$ or relate$)).tw. |
| 20 | ((patient$ or client$ or consumer$ or recipient$ or subject$ or service user$ or care?giver$ or care giver$ or carer$ or famil$) adj3 (attitude$ or trust)).tw. |
| 21 | ((patient$ or client$ or consumer$ or recipient$ or subject$ or service user$ or care?giver$ or care giver$ or carer$ or famil$) adj3 (educat$ or inform$ or train$)).tw. |
| 22 | ((patient$ or client$ or consumer$ or recipient$ or subject$ or service user$ or care?giver$ or care giver$ or carer$ or famil$) adj3 (counsel$ or advise or advice)).tw. |
| 23 | ((patient$ or client$ or consumer$ or recipient$ or subject$ or service user$ or care?giver$ or care giver$ or carer$ or famil$) adj3 (participat$ or shar$ or joint or empower$ or involve$ or engage$)).tw. |
| 24 | ((patient$ or client$ or consumer$ or recipient$ or subject$ or service user$ or care?giver$ or care giver$ or carer$ or famil$) adj3 (decision$ or choice$ or preference$)).tw. |
| 25 | ((patient$ or client$ or consumer$ or recipient$ or subject$ or service user$ or care?giver$ or care giver$ or carer$ or famil$) adj3 (directive$ or care planning)).tw. |
| 26 | decision aid$.tw. |
| 27 | (cue card$ or prompt$ or checklist$).tw. |
| 28 | (pre-consultation$ or preconsultation$).tw. |
| 29 | feedback form$.tw. |
| 30 | ((patient$ or client$ or consumer$ or recipient$ or subject$ or service user$ or care?giver$ or care giver$ or carer$ or famil$) adj3 (internet or computer$)).tw. |
| 31 | ((patient$ or client$ or consumer$ or recipient$ or subject$ or service user$ or care?giver$ or care giver$ or carer$ or famil$) adj3 (pamphlet$ or leaflet$ or diary or diaries or sheet$ or brochure$ or booklet$)).tw. |
| 32 | or/7-31 |
|  | |
| Phenomenon of Interest - context: Primary care | |
| 33 | General Practice/ |
| 34 | Family Practice/ |
| 35 | exp Primary Health Care/ |
| 36 | General Practitioner/ |
| 37 | Physicians, Family/ |
| 38 | Community Care/ |
| 39 | Community Health Nursing/ |
| 40 | Community Health Services/ |
| 41 | Community Pharmacy Services/ |
| 42 | Home Care Services/ |
| 43 | general practice$.tw. |
| 44 | (family pract$ or family medicine).tw. |
| 45 | (primary care or primary health care or primary healthcare or primary medical care).tw. |
| 46 | (community adj (health or healthcare)).tw. |
| 47 | (community?based or community based or community care).tw. |
| 48 | (general practitioner$ or general physician$ or GP$ or GPSI or GPwSI).tw. |
| 49 | (practitioner$ adj3 special interest$).tw. |
| 50 | (family practitioner$ or family physician$ or family doctor$).tw. |
| 51 | (primary care practitioner$ or primary care physician$).tw. |
| 52 | (primary healthcare team$ or primary health care team$ or primary medical care team$).tw. |
| 53 | (practice nurse$ or practice manager$ or practice receptionist$ or practice pharmacist$).tw. |
| 54 | community pharmacy service$.tw. |
| 55 | home care service$.tw. |
| 56 | (primary care or primary health care or general practice or family practice or family medicine).jx. |
| 57 | or/33-56 |
|  |  |
| Design / Research type: Qualitative research | |
| 58 | exp Qualitative Research/ |
| 59 | Grounded Theory/ |
| 60 | Constant Comparative Method/ |
| 61 | Content Analysis/ |
| 62 | Discourse Analysis/ |
| 63 | Conversation Analysis/ |
| 64 | Thematic Analysis/ |
| 65 | Audio recording/ |
| 66 | Participant observation/ |
| 67 | Ethnography/ |
| 68 | Field study/ |
| 69 | Semi-structured interview/ |
| 70 | Unstructured interview/ |
| 71 | Focus Groups/ |
| 72 | Narratives/ |
| 73 | Purposive sample/ |
| 74 | Theoretical sample/ |
| 75 | qualitative.tw. |
| 76 | (grounded adj2 (theor$ or study or studies or research or analysis)).tw. |
| 77 | constant comparative method$.tw. |
| 78 | content analysis.tw. |
| 79 | (conversation analysis or discourse analysis).tw. |
| 80 | ((theme or thematic or themes) adj2 analysis).tw. |
| 81 | (audio?recorded or audio recorded or audio?recording or audio recording).tw. |
| 82 | (tape recorded or tape recording).tw. |
| 83 | (video?recorded or video recorded or video?recording or video recording).tw. |
| 84 | participant observation.tw. |
| 85 | ((ethnographic or ethnological or ethnology) adj2 (research or study)).tw. |
| 86 | ethnonursing.tw. |
| 87 | (naturalistic adj2 field study).tw. |
| 88 | field notes.tw. |
| 89 | (semi-structured adj2 (question$ or interview$)).tw. |
| 90 | ((unstructured or un-structured) adj2 (question$ or interview$)).tw. |
| 91 | focus group$.tw. |
| 92 | narrative analysis.tw. |
| 93 | ((theoretical or purposive) adj1 (sample or sampling)).tw. |
| 94 | hermeneutic.tw. |
| 95 | ((phenomenology or phenomenological) adj2 research).tw. |
| 96 | (key informant adj2 (question$ or interview$)).tw. |
| 97 | Life world$.tw. |
| 98 | lived experience.tw. |
| 99 | ((life or womens) adj2 (story or stories)).tw. |
| 100 | qualitative.jx. |
| 101 | or/58-100 |
|  |  |
| Total | |
| 102 | 6 and 32 and 57 and 101 |
| 103 | limit 102 to English language |

**Search strategy for the EBSCO platform**

| Sample: Older people | |
| --- | --- |
| 1 | MH "aging+" |
| 2 | TI (("old" or "older" or "aging" or "senior") N2 ("person" or "persons" or "people" or "adult*" or "subject*" or "patient*" or "consumer*")) OR AB (("old" or "older" or "aging" or "senior") N2 ("person" or "persons" or "people" or "adult*" or "subject*" or "patient*" or "consumer*")) |
| 3 | TI (("old" or "older" or "aging" or "senior") N2 ("service user*")) OR AB (("old" or "older" or "aging" or "senior") N2 ("service user*")) |
| 4 | TI (("old" or "older" or "aging" or "senior") N2 ("men" or "male" or "males" or "women" or "female*")) OR AB (("old" or "older" or "aging" or "senior") N2 ("men" or "male" or "males" or "women" or "female*")) |
| 5 | TI ("late life" or "elder*" or "old* age" or "geriatric" or "seniors") OR AB ("late life" or "elder*" or "old* age" or "geriatric" or "seniors") |
| 6 | S1 OR S2 OR S3 OR S4 OR S5 |
|  | |
| Phenomenon of Interest - domain: Patient verbal communication in interactions | |
| 7 | MH "Communication" |
| 8 | MH "Physician-Patient Relations" |
| 9 | MH "Patient Education" |
| 10 | MH "Consumer Participation" |
| 11 | MH "Patient Centered Care" |
| 12 | MH "Decision Making" |
| 13 | MH "Advance Directives" |
| 14 | MH "Decision Support Techniques" |
| 15 | TI (("patient*" or "client*" or "consumer*" or "recipient*" or "subject*" or "service user*" or "care#giver*" or "care giver*" or "carer*" or "famil*") N2 ("communicat*")) OR AB (("patient*" or "client*" or "consumer*" or "recipient*" or "subject*" or "service user*" or "care#giver*" or "care giver*" or "carer*" or "famil*") N2 ("communicat*")) |
| 16 | TI (("patient*" or "client*" or "consumer*" or "recipient*" or "subject*" or "service user*" or "care#giver*" or "care giver*" or "carer*" or "famil*") N2 ("verbal" or "talk" or "discourse*" or "discussion*" or "conversation*")) OR AB (("patient*" or "client*" or "consumer*" or "recipient*" or "subject*" or "service user*" or "care#giver*" or "care giver*" or "carer*" or "famil*") N2 ("verbal" or "talk" or "discourse*" or "discussion*" or "conversation*")) |
| 17 | TI (("patient*" or "client*" or "consumer*" or "recipient*" or "subject*" or "service user*" or "care#giver*" or "care giver*" or "carer*" or "famil*") N2 ("interact*" or "relation*" or "relate*")) OR AB (("patient*" or "client*" or "consumer*" or "recipient*" or "subject*" or "service user*" or "care#giver*" or "care giver*" or "carer*" or "famil*") N2 ("interact*" or "relation*" or "relate*")) |
| 18 | TI (("patient*" or "client*" or "consumer*" or "recipient*" or "subject*" or "service user*" or "care#giver*" or "care giver*" or "carer*" or "famil*") N2 ("attitude*" or "trust")) OR AB (("patient*" or "client*" or "consumer*" or "recipient*" or "subject*" or "service user*" or "care#giver*" or "care giver*" or "carer*" or "famil*") N2 ("attitude*" or "trust")) |
| 19 | TI (("patient*" or "client*" or "consumer*" or "recipient*" or "subject*" or "service user*" or "care#giver*" or "care giver*" or "carer*" or "famil*") N2 ("educat*" or "inform*" or "train*")) OR AB (("patient*" or "client*" or "consumer*" or "recipient*" or "subject*" or "service user*" or "care#giver*" or "care giver*" or "carer*" or "famil*") N2 ("educat*" or "inform*" or "train*")) |
| 20 | TI (("patient*" or "client*" or "consumer*" or "recipient*" or "subject*" or "service user*" or "care#giver*" or "care giver*" or "carer*" or "famil*") N2 ("counsel*" or "advise" or "advice")) OR AB (("patient*" or "client*" or "consumer*" or "recipient*" or "subject*" or "service user*" or "care#giver*" or "care giver*" or "carer*" or "famil*") N2 ("counsel*" or "advise" or "advice")) |
| 21 | TI (("patient*" or "client*" or "consumer*" or "recipient*" or "subject*" or "service user*" or "care#giver*" or "care giver*" or "carer*" or "famil*") N2 ("participat*" or "shar*" or "joint" or "empower*" or "involve*" or "engage*")) OR AB (("patient*" or "client*" or "consumer*" or "recipient*" or "subject*" or "service user*" or "care#giver*" or "care giver*" or "carer*" or "famil*") N2 ("participat*" or "shar*" or "joint" or "empower*" or "involve*" or "engage*")) |
| 22 | TI (("patient*" or "client*" or "consumer*" or "recipient*" or "subject*" or "service user*" or "care#giver*" or "care giver*" or "carer*" or "famil*") N2 ("decision*" or "choice*" or "preference*")) OR AB (("patient*" or "client*" or "consumer*" or "recipient*" or "subject*" or "service user*" or "care#giver*" or "care giver*" or "carer*" or "famil*") N2 ("decision*" or "choice*" or "preference*")) |
| 23 | TI (("patient*" or "client*" or "consumer*" or "recipient*" or "subject*" or "service user*" or "care#giver*" or "care giver*" or "carer*" or "famil*") N2 ("directive*" or "care planning")) OR AB (("patient*" or "client*" or "consumer*" or "recipient*" or "subject*" or "service user*" or "care#giver*" or "care giver*" or "carer*" or "famil*") N2 ("directive*" or "care planning")) |
| 24 | TI "decision aid*" OR AB "decision aid*" |
| 25 | TI ("cue card*" or "prompt*" or "checklist*") OR AB ("cue card*" or "prompt*" or "checklist*") |
| 26 | TI ("pre-consultation*" or "pre#consultation*") OR AB ("pre-consultation*" or "pre#consultation*") |
| 27 | TI "feedback form*" OR AB "feedback form*" |
| 28 | TI (("patient*" or "client*" or "consumer*" or "recipient*" or "subject*" or "service user*" or "care#giver*" or "care giver*" or "carer*" or "famil*") N2 ("internet" or "computer*")) OR AB (("patient*" or "client*" or "consumer*" or "recipient*" or "subject*" or "service user*" or "care#giver*" or "care giver*" or "carer*" or "famil*") N2 ("internet" or "computer*")) |
| 29 | TI (("patient*" or "client*" or "consumer*" or "recipient*" or "subject*" or "service user*" or "care#giver*" or "care giver*" or "carer*" or "famil*") N2 ("pamphlet*" or "leaflet*" or "diary" or "diaries" or "sheet*" or "brochure*" or "booklet*")) OR AB (("patient*" or "client*" or "consumer*" or "recipient*" or "subject*" or "service user*" or "care#giver*" or "care giver*" or "carer*" or "famil*") N2 ("pamphlet*" or "leaflet*" or "diary" or "diaries" or "sheet*" or "brochure*" or "booklet*")) |
| 30 | S7 OR S8 OR S9 OR S10 OR S11 OR S12 OR S13 OR S14 OR S15 OR S16 OR S17 OR S18 OR S19 OR S20 OR S21 OR S22 OR S23 OR S24 OR S25 OR S26 OR S27 OR S28 OR S29 |
|  | |
| Phenomenon of Interest - context: Primary care | |
| 31 | MH "Family Practice" |
| 32 | MH "Primary Health Care" |
| 33 | MH "Physicians, Family" |
| 34 | MH "Community Health Centers" |
| 35 | MH "Community Health Nursing" |
| 36 | MH "Community Health Services" |
| 37 | MH "Medication Management" |
| 38 | MH "Home Health Care" |
| 39 | TI "general practice*" OR AB "general practice*" |
| 40 | TI ("family pract*" or "family medicine") OR AB ("family pract*" or "family medicine") |
| 41 | TI ("primary care" or "primary health care" or "primary healthcare" or "primary medical care") OR AB ("primary care" or "primary health care" or "primary healthcare" or "primary medical care") |
| 42 | TI ("community" W0 ("health" or "healthcare")) OR AB ("community" W0 ("health" or "healthcare")) |
| 43 | TI ("community#based" or "community based" or "community care") OR AB ("community#based" or "community based" or "community care") |
| 44 | TI ("general practitioner*" or "general physician*" or "GP*" or "GPSI" or "GPwSI") OR AB ("general practitioner*" or "general physician*" or "GP*" or "GPSI" or "GPwSI") |
| 45 | TI ("practitioner*" N2 "special interest*") OR AB ("practitioner*" N2 "special interest*") |
| 46 | TI ("family practitioner*" or "family physician*" or "family doctor*") OR AB ("family practitioner*" or "family physician*" or "family doctor*") |
| 47 | TI ("primary care practitioner*" or "primary care physician*") OR AB ("primary care practitioner*" or "primary care physician*") |
| 48 | TI ("primary healthcare team*" or "primary health care team*" or "primary medical care team*") OR AB ("primary healthcare team*" or "primary health care team*" or "primary medical care team*") |
| 49 | TI ("practice nurse*" or "practice manager*" or "practice receptionist*" or "practice pharmacist*") OR AB ("practice nurse*" or "practice manager*" or "practice receptionist*" or "practice pharmacist*") |
| 50 | TI "community pharmacy service*" OR AB "community pharmacy service*" |
| 51 | TI "home care service*" OR AB "home care service*" |
| 52 | SO ("primary care" or "primary health care" or "general practice" or "family practice" or "family medicine") |
| 53 | S31 OR S32 OR S33 OR S34 OR S35 OR S36 OR S37 OR S38 OR S39 OR S40 OR S41 OR S42 OR S43 OR S44 OR S45 OR S46 OR S47 OR S48 OR S49 OR S50 OR S51 OR S52 |
|  |  |
| Design / Research type: Qualitative research | |
| 54 | MH "Qualitative Studies+" |
| 55 | MH "Grounded Theory" |
| 56 | MH "Constant Comparative Method" |
| 57 | MH "Content Analysis" |
| 58 | MH "Discourse Analysis" |
| 59 | MH "Thematic Analysis" |
| 60 | MH "Audiorecording" |
| 61 | MH "Participant observation" |
| 62 | MH "Ethnographic Research" |
| 63 | MH "Field studies" |
| 64 | MH "Semi-structured interview" |
| 65 | MH "Unstructured interview" |
| 66 | MH "Focus Groups" |
| 67 | MH "Narratives" |
| 68 | MH "Purposive sample" |
| 69 | MH "Theoretical sample" |
| 70 | TI "qualitative" OR AB "qualitative" |
| 71 | TI ("grounded" N1 ("theor*" or "study" or "studies" or "research" or "analysis")) OR AB ("grounded" N1 ("theor*" or "study" or "studies" or "research" or "analysis")) |
| 72 | TI "constant comparative method*" OR AB "constant comparative method*" |
| 73 | TI "content analysis" OR AB "content analysis" |
| 74 | TI ("conversation analysis" or "discourse analysis") OR AB ("conversation analysis" or "discourse analysis") |
| 75 | TI (("theme" or "thematic" or "themes") N1 "analysis") OR AB (("theme" or "thematic" or "themes") N1 "analysis") |
| 76 | TI ("audio#recorded" or "audio recorded" or "audio#recording" or "audio recording") OR AB ("audio#recorded" or "audio recorded" or "audio#recording" or "audio recording") |
| 77 | TI ("tape recorded" or "tape recording") OR AB ("tape recorded" or "tape recording") |
| 78 | TI ("video#recorded" or "video recorded" or "video#recording" or "video recording") OR AB ("video#recorded" or "video recorded" or "video#recording" or "video recording") |
| 79 | TI "participant observation" OR AB "participant observation" |
| 80 | TI (("ethnographic" or "ethnological" or "ethnology") N1 ("research" or "study")) OR AB (("ethnographic" or "ethnological" or "ethnology") N1 ("research" or "study")) |
| 81 | TI "ethnonursing" OR AB "ethnonursing" |
| 82 | TI ("naturalistic" N1 "field study") OR AB ("naturalistic" N1 "field study") |
| 83 | TI "field notes" OR AB "field notes" |
| 84 | TI ("semi-structured" N1 ("question*" or "interview*")) OR AB ("semi-structured" N1 ("question*" or "interview*")) |
| 85 | TI (("unstructured" or "un-structured") N1 ("question*" or "interview*")) OR AB (("unstructured" or "un-structured") N1 ("question*" or "interview*")) |
| 86 | TI "focus group*" OR AB "focus group*" |
| 87 | TI "narrative analysis" OR AB "narrative analysis" |
| 88 | TI (("theoretical" or "purposive") N0 ("sample" or "sampling")) OR AB (("theoretical" or "purposive") N0 ("sample" or "sampling")) |
| 89 | TI "hermeneutic" OR AB "hermeneutic" |
| 90 | TI (("phenomenology" or "phenomenological") N1 "research") OR AB (("phenomenology" or "phenomenological") N1 "research") |
| 91 | TI ("key informant" N1 ("question*" or "interview*")) OR AB ("key informant" N1 ("question*" or "interview*")) |
| 92 | TI "Life world*" OR AB "Life world*" |
| 93 | TI "lived experience" OR AB "lived experience" |
| 94 | TI (("life" or "womens") N1 ("story" or "stories")) OR AB (("life" or "womens") N1 ("story" or "stories")) |
| 95 | SO "qualitative" |
| 96 | S54 OR S55 OR S56 OR S57 OR S58 OR S59 OR S60 OR S61 OR S62 OR S63 OR S64 OR S65 OR S66 OR S67 OR S68 OR S69 OR S70 OR S71 OR S72 OR S73 OR S74 OR S75 OR S76 OR S77 OR S78 OR S79 OR S80 OR S81 OR S82 OR S83 OR S84 OR S85 OR S86 OR S87 OR S88 OR S89 OR S90 OR S91 OR S92 OR S93 OR S94 OR S95 |
|  |  |
| Total | |
| 97 | S6 and S30 and S53 and S96 |
| 98 | limit S97 to English language |
