# Supplementary material for: Older Patients' Verbal Communication in Interactions With Primary Care Staff: A Qualitative Systematic Review and Meta‐Ethnography
Source: Health Expect. 2025 Aug 28;28(5):e70355. doi: 10.1111/hex.70355 (PMC12392129; doi:10.1111/hex.70355)
Supplement: Supplementary file 2 — Supplementary Material 2: Data Extraction Template. [file HEX-28-e70355-s002.docx]

## **DATA EXTRACTION TEMPLATE**

| Reference ID |  |
| --- | --- |
| Author(s) |  |
| Publication year |  |
| Title |  |
| Extracted by |  |
| Date extracted on |  |

**General**

| Study type / design |  |
| --- | --- |
| Discipline(s) |  |
| Study location / Country |  |
| Setting |  |
| Research question(s) / Objective(s) |  |

**Qualitative participants**

| Population |  |
| --- | --- |
| Number of participants |  |
| Age  Average and/or range |  |
| Gender |  |
| Ethnicity |  |
| Education / Socioeconomic status |  |
| Recruitment / sampling Inclusion and exclusion criteria |  |
| Response  Response rates, differences between responders and non-responders |  |

**Qualitative data collection and analysis**

| Method of data collection |  |
| --- | --- |
| Method of data analysis |  |
| Theoretical underpinning(s) |  |
| Presentation of results |  |
| Trustworthiness |  |

**Qualitative results**

Extract in detail, using author’s own words (in quotation marks with page references), focusing on results relevant to the research question(s)

Do not include quotes from the study participants. Refer primarily to the ‘headline’ results / themes. However, other results should be noted where these are especially relevant. Key quotes may be present in the abstract or discussion. If you make your own interpretations of the data, record these but clearly label as your interpretation.

| Theme / Concept #1 |  |
| --- | --- |
| Theme / Concept #2 |  |
| Theme / Concept #3 |  |
| Theme / Concept #4 |  |
| Theme / Concept #5 |  |

**Other**

| Important limitations |  |
| --- | --- |
| Comments |  |

**Quality**

| Are the aims and objectives clearly stated?  Consider the goal of the research, its importance and its relevance | | Yes / No / To some extent |
| --- | --- | --- |
| Is the design clearly specified and appropriate?  Consider whether the design has been justified and whether it is appropriate given the aims of the research | | Yes / No / To some extent |
| Is the recruitment strategy and method of data collection clearly specified and appropriate?  Consider how participants were selected, how data were collected, whether this approach has been justified, whether it is appropriate, and whether ethical issues have been considered | | Yes / No / To some extent |
| Is the method of analysis clearly specified and appropriate?  Consider the depth of the description of the analysis, whether it is clear how the findings were derived, and whether researcher bias has been taken into account | | Yes / No / To some extent |
| Do the researchers display enough data to support their interpretations and conclusions?  Consider whether evidence is presented for and against the researchers’ argument(s), and whether the credibility of their findings are discussed | | Yes / No / To some extent |
| Rating | 1. Key paper 2. Satisfactory paper 3. Unsure 4. Fatally flawed 5. Irrelevant to the research question | |
| If ‘Unacceptable,’ why? |  | |

**References**

| Relevant references  For inclusion or background |  |
| --- | --- |
| Relevant citations  For inclusion or background |  |
